# High prevalence of hip and groin problems in professional ice hockey players, regardless of playing position

**DOI:** 10.1007/s00167-019-05787-7

**Published:** 2019-11-16

**Authors:** Tobias Wörner, Kristian Thorborg, Frida Eek

**Affiliations:** 1 Department of Health Sciences, Lund University, Box 157, 221 00 Lund, Sweden; 2 Sports Orthopedic Research Center–Copenhagen, Department of Orthopaedic Surgery, Copenhagen University Hospital, Amager-Hvidovre, Denmark

**Keywords:** Ice hockey, Epidemiology, Groin pain, Hip pain, Hip arthroscopy

## Abstract

**Purpose:**

The prevalence of hip and groin problems in professional male ice hockey is unknown and suspected to differ between playing positions. The purpose of this study was to explore potential differences in the seasonal prevalence of hip and groin problems between playing positions in male elite ice hockey players and to explore the relationship between symptom duration and hip and groin function at the beginning of the new season.

**Methods:**

Male ice hockey players [*n* = 329 (92 goalkeepers, 93 defensemen, 144 forwards), Mean age (SD): 24 (5)] from the professional leagues in Sweden responded to an online survey. The survey assessed presence of hip and groin problems (time loss and non-time loss) and symptom duration (categorized into 0, 1–6, or > 6 weeks) in the previous season, and current self-reported hip and groin function (Copenhagen Hip and Groin Outcome Score).

**Results:**

During the previous season, 175 players (53.2%) had experienced hip and groin problems. Non time loss problems were experienced by 158 (48%) and time loss problems were experienced by 97 (29.5%) players. No significant differences between playing positions were found. Self-reported function differed significantly between players with different symptom duration and more disability was reported among players with longer symptom duration (*p* ≤ 0.002).

**Conclusion:**

Regardless of playing position, hip and groin problems were prevalent in male ice hockey players. Players with hip and groin problems during the previous season had significantly worse hip and groin function in the beginning of the new season, and longer symptom duration was associated with more disability.

**Level of evidence:**

III

## Introduction

Ice hockey is one of the most popular sports in Sweden and injury risks are reportedly high [1]. Despite the popularity of the sport and large numbers of injuries, the last epidemiological studies from Sweden were published more than 25 years ago [20, 24], and recent studies focus predominantly on concussion and dental health [10, 11, 23]. Epidemiological knowledge regarding common musculoskeletal injuries such as hip and groin pain in Swedish ice hockey is thus currently lacking.

North American studies report groin injuries to be common in ice hockey [5, 15]. In the National Hockey League (NHL), approximately 10% of all hip and groin injuries are reported to be intra-articular [8], and high numbers of ice hockey players undergo arthroscopic surgery for the treatment of femoroacetabular impingement syndrome (FAIS) [19, 22]. While forwards demonstrate the highest injury rates among collegiate athletes [5], some studies suggest that goalkeepers may be at higher risk for hip and groin injuries [5, 8, 31]. However, there is reason to believe that current methods of reporting hip and groin injuries in ice hockey do not capture the nature of these problems appropriately. In the existing literature, hip and groin injuries in professional ice hockey are defined as incidence of time loss or medical attention injuries [5, 7, 8, 28]. This definition may, however, underestimate the burden of hip and groin problems in ice hockey, where injuries often present as longstanding overuse problems, not necessarily leading to time loss [15]. It has been suggested that the reporting of prevalence as opposed to incidence may be a more appropriate measure to report overuse injuries [2, 4] and that hip and groin pain should be investigated beyond the time loss definition of injury [29]. Following these suggestions, recent research in soccer revealed that groin problems are much more prevalent than previously thought and that a time-loss definition would have only captured one-third of the reported problems [13]. Furthermore, hip and groin problems in one season appear to be associated with remaining functional impairments in the beginning of the new season [27]. Similar studies in ice hockey players, describing the prevalence and functional consequences of hip and groin problems, may provide a foundation for the planning and evaluation of future preventive efforts.

The aim of this study was to explore potential differences in the seasonal prevalence of hip and groin problems between playing positions in male elite ice hockey players. Furthermore, the relation between symptom duration during the previous season and hip and groin function in the beginning of the new season was analyzed. It was expected to observe (1) a high general prevalence of hip and groin problems (2) that goalkeepers were more affected than other players and (3) that players with problems during the previous season experienced impaired hip and groin function in the beginning of the new season.

## Materials and methods

Using a cross-sectional design, this study surveyed elite ice hockey players in Sweden regarding the experience of hip and groin problems during the previous season as well as self-reported hip and groin function in the beginning of the new season. At the beginning of the 2017–2018 season, all male, senior and junior players playing in the most competitive leagues in Sweden were invited for participation in an online survey. Participants received written information about the study. The survey was anonymous, and response was optional. Therefore, response to the survey was considered informed consent. The study was approved by the Ethics Committee at Lund University (Dnr 2017/483).

### Participants and recruitment

All active male players in the professional Swedish ice-hockey leagues [Swedish Hockey League (SHL); Hockey Allsvenskan; Swedish Junior Hockey league (J-20 SuperElit)], being able to understand Swedish or English, were eligible to participate in the study. Data collection for goalkeepers and three full teams was performed within the frame of separate, prospective studies. In an attempt to recruit additional defenders and forwards and allow comparison of playing positions, an open invitation for participation as well as the link to the survey was provided to medical representatives of all remaining professional clubs in respective leagues. Additionally, medical officials were asked to distribute the invitation and survey link to players. Furthermore, an invitation and link to the survey were posted on the home page of the players union (SICO).

### Questionnaire survey

The web-based questionnaire survey (administered in both Swedish and English) consisted of questions regarding player demographics and background information such as level of play (league), position, and years of playing professional ice hockey. Prevalence of hip and groin problems was assessed by asking players the following: (1) “did you, at any occasion during the previous season, have an injury, pain or symptoms in the hip/groin region that completely prevented you from participating in training/match play?” (time loss problem) and (2) “did you, at any occasion during the previous season, have an injury, pain or symptoms in the hip/groin region that affected your performance during training/match play (non-time loss problem)”. Furthermore, players were asked to report duration (in weeks) of hip and groin problems and if they had a history hip and groin surgery, which was then categorized into (a) hip arthroscopy for FAIS or (b) other groin surgery such as inguinal repairs or adductor tenotomy. Self-reported hip and groin function was assessed by the Copenhagen Hip and Groin Outcome Score (HAGOS) [26]. The HAGOS questionnaire consists of 36 items evaluating hip and groin function in the previous week in six domains: pain, symptoms, activity of daily living, sport, physical activity (PA), and quality of life (QOL).

### Statistical analysis

#### Data management

Player positions were categorized as either goalkeepers, defenders, or forwards. Duration of hip and groin problems was categorized as zero weeks (‘no hip and groin problems’) or longest duration of continuous hip and groin problems (either time loss or non-time loss) of 1–6 weeks (‘hip and groin problems’) or > 6 weeks (‘longstanding hip and groin problems’). Scores for each HAGOS subscale domain were computed and transformed to a 0–100 scale, where zero represents extreme hip and/or groin problems and 100 represents no hip and/or groin problems. In the case of ≤ 1 missing value for items within the subdomain “Participation in Physical Activities” and ≤ 2 missing values for items within the other subdomains, mean scores of the respective domain were imputed. If more values were missing, no sub-score for the domain was computed.

#### Analysis

Data analysis was performed using SPSS Statistics 23 (IBM Software). Group statistics were presented in the form of frequencies [percentage (95%CI)] for nominal data, and median [interquartile range (IQR)] or means [standard deviations (SD)] for ordinal data or ratio scale data. Comparisons between player positions regarding the prevalence of hip and groin problems were performed by Chi-square tests. HAGOS scores at the start of the new season were compared between player positions as well as between players with no hip and groin problems (zero recorded weeks of symptoms), hip and groin problems (1–6 weeks’ symptom duration), and longstanding hip and groin problems (> 6 weeks of symptom duration) during the previous season through Kruskal–Wallis tests with pair-wise post hoc comparison.

No sample size calculation was performed prior to data collection due to the descriptive nature of the study—the aim was to include as many players as possible from the target population.

## Results

**Fig. 1:**
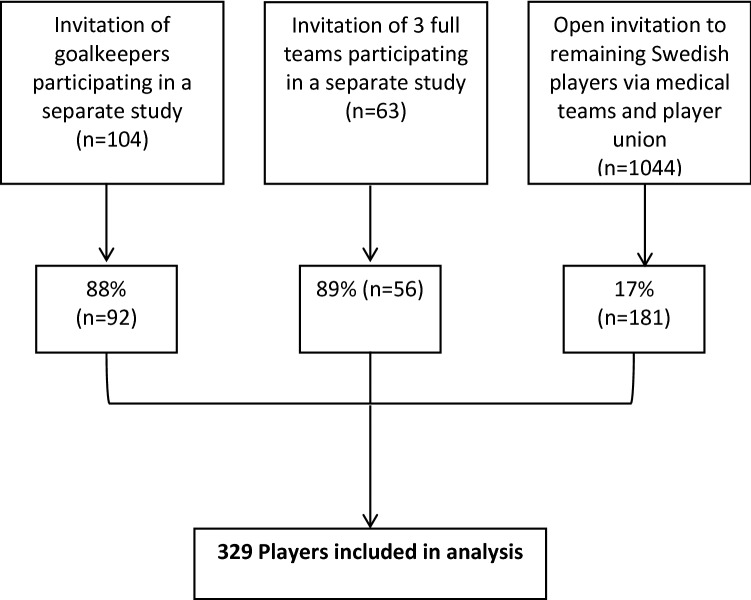
Flow of participants into the study

The final sample consisted of 329 players. Participant study flow is summarized in Fig. 1. Demographics are provided in Table 1. All 329 players provided complete data on seasonal prevalence and all, but one player provided responses to the HAGOS questionnaire.

**Table 1:** Demographics of study sample and target population

	Goalkeepers sample (*n* = 92)	Goalkeepers population (*n* = 138)	Defenders sample (*n* = 93)	Defenders population (*n* = 452)	Forwards sample (*n* = 144)	Forwards population (*n* = 796)	Sample total (*n* = 329)	Population total (*n* = 1386)
Age in years [Mean (SD)]	21.8 (5.0)	21.4 (5.1)	24.8 (5.3)	21.5 (5.1)	24.8 (5.0)	21.8 (5.0)	24 (5.3)	22 (5.1)
Height (cm) [Mean (SD)]	185.9 (5.1)	184.6 (5.1)	184.8 (5.8)	182.6 (10.2)	183.1 (5.4)	181.9 (5.7)	184 (5.6)	182 (7.5)
Weight (kg) [Mean (SD)]	85.2 (6.2)	81.5 (7.1)	87.5 (6.4)	82.4 (9.3)	86.3 (7.5	81.7 (7.8)	86.4 (6.9)	82 (8.2)
Playing ice hockey (years) [Mean (SD)]	5.3 (5.4)	–	9.6 (6.9)	–	9.7 (6.6)	–	8.5 (6.7)	–
Playing level [*n* (%)]								
SHL	26 (28.3)	40 (29.0)	44 (47.3)	134 (29.6)	73 (50.7)	232 (29.1)	143 (43.5)	406 (29.3)
Hockey allsvenskan	26 (28.3)	47 (34.1)	37 (39.8)	125 (27.7)	54 (37.5)	243 (30.5)	117 (35.6)	415 (29.9)
J-20 SuperElit	40 (43.5)	51 (37.0)	12 (12.9)	193 (42.7)	17 (11.8)	321 (40.3)	69 (21.0)	565 (40.8)

Data on the total population retrieved from the Swedish Ice Hockey Association (https://stats.swehockey.se)

*NB* “Population total” represents all players listed as potential players by their clubs before the season and differs from the total number of players actually starting the season (see Fig. 1), *SD* standard deviation, *SHL* Swedish Hockey League, *J-20* under 20 years

### Comparison of playing positions

No significant differences in the prevalence of hip and groin problems were found between goalkeepers, defensemen, and forwards (Table 2).

**Table 2:** Seasonal prevalence of hip and groin problems by player position (*n* = 329)

	Goalkeepers (*n* = 92)	Defenders (*n* = 93)	Forward (*n* = 144)	*P* value*
Any problem (TL/NTL) % (95% CI)	53.3 (43–63)	53.8 (44–64)	52.8 (44–61)	ns
TL problem % (95% CI)	22.8 (15–32)	30.1 (21–40)	33.3 (26–41)	ns
Duration of TL problem in weeks [Median (IQR)]	1.5 (1–4)	3 (1–5.3)	2 (1–2.75)	ns
NTL problem % (95% CI)	48.9 (39–59)	48.4 (39–58)	47.2 (39–55)	ns
Duration of NTL problem in weeks [Median (IQR)]	2 (1–4)	3 (1–8)	3 (1.5–5.5)	ns

*TL* time loss, *NTL* non time loss, *IQR* interquartile range, *CI* confidence interval

*Chi-square test for any/TL-/NTL-problems; Kruskal–Wallis test for duration (significance level .05)

### Seasonal prevalence and duration of time loss and non-time loss injuries

In total, 175 players (53.2%) (*n* = 175) reported having hip and groin problems during the previous season. Hip and groin problems, classified as time-loss injuries, were reported by 97 players [29.5% (CI 95% 25–35%)] of all players. Affected players reported a median time loss of 2 weeks (IQR: 1–4). Almost half of the players [*n* = 158; 48% (CI 95% 43–53%)] reported having had non time loss injuries of the hip and groin during the previous season. Median duration of non-time loss injuries was 3 weeks (IQR: 1–4). A history of hip and groin surgery was reported by 26 players [7.9% (CI 95% 5–11%)]. Two-thirds of these surgeries were hip arthroscopies for FAIS (*n* = 17) and one third were other groin surgeries such as tenotomies or inguinal hernia repairs (*n* = 9).

### Self-reported hip and groin function in the beginning of the season

**Fig. 2:**
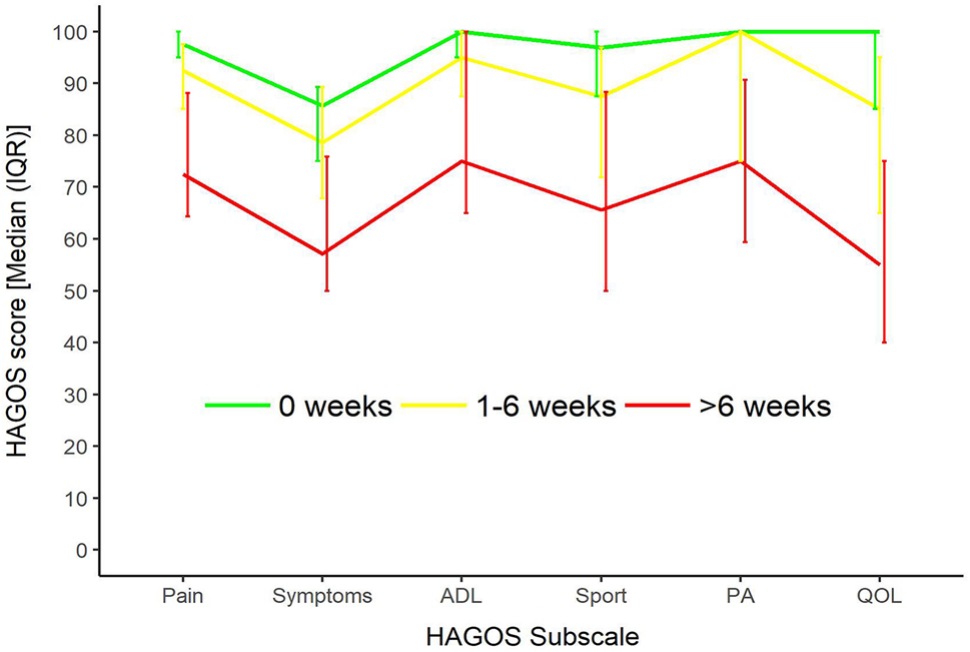
HAGOS scores in the beginning of the new season in players with different hip and groin pain durations during the previous season. *HAGOS* hip and groin outcome score, *IQR* inter quartal range, *ADL* activities of daily living, *PA* physical activity, *QOL* quality of life. All groups differed significantly from each other on all HAGOS subscales (*p* ≤ 0.002 [Kruskal–Wallis test (significance level 0.05)]

Self-reported hip and groin function in the beginning of the new season differed significantly between all symptom duration groups (0, 1–6, and > 6 weeks) (*p* ≤ 0.002). The direction of these significant differences indicates that players with longer symptom duration in the previous season reported more disability in the beginning of the new season (Fig. 2).

## Discussion

The present study found that hip and groin problems were prevalent in professional ice hockey players in Sweden regardless of playing position. Over half (53.2%) of the players had experienced some hip and groin problems during the previous season. Forty-eight percent of players reported a problem that affected their performance, and 28% of players reported inability to train or play because of them. Players with a history of hip and groin problems during the previous season had significantly worse hip and groin function at the start of the following season. Longer duration of hip problems was associated with more impairments in self-reported function at the beginning of the new season.

This is the first study measuring the burden of hip and groin problems in professional ice hockey reporting period prevalence as opposed to incidence rates, as per previous studies [5, 15]. While results of this study cannot be directly compared to previous findings, they provide a novel, and arguably more appropriate, perspective on the burden of hip and groin problems in this group of athletes [2]. Half of all players participating in the study experienced a hip and groin problem affecting their performance (non-time loss) and almost one third of all participating players were side-lined because of them (time loss). Studies on collegiate ice hockey players in North America report that over 50% of hip and groin injuries are non-time loss injuries [5, 6, 15]. However, according to the National Collegiate Athletic Association, non-time loss injuries are recorded only after reports to team clinicians or athletic trainers [16]. Nonetheless, it is not common that athletes avoid seeking medical attention for injuries out of fear of being side-lined [3, 17]. Arguably, current literature may, therefore, fail to include a large proportion of hip and groin problems which do not lead to time loss, but nevertheless impair performance and have long-lasting impact on hip and groin function.

Players with hip and groin problems during the previous season reported significantly worse hip and groin function at the beginning of the new season compared to players who did not report prior hip and groin problems. This indicates that hip and groin problems have long-lasting impact on players and may not resolve despite off-season breaks and pre-season training. Among players that reported symptoms during the previous season, players with longstanding symptoms (>6 weeks duration) presented with most severe impairments in self-reported hip and groin function at the beginning of the new season. These findings align with previous research on soccer players [27], indicating that emerging or existing hip and groin problems should be identified and addressed as early as possible to prevent long-lasting functional impairment (secondary prevention), as proposed in a recent study on soccer players [32]. A potential tool to monitor and identify players with existing or emerging hip and groin problems is the five-second squeeze test (5SST) [25]. The 5SST involves maximal bilateral adduction for 5 s against an assessor’s forearm, followed by a quantification of experienced groin pain on a numeric rating scale from zero (no pain) to 10 (maximal pain). This test has been shown to indicate self-reported sporting function as well as hip adduction and abduction strength in ice hockey players [33] and could potentially be used to identify players that may benefit from targeted interventions.

No statistically significant differences were found in the seasonal prevalence of hip and groin problems, self-reported function, or history of hip arthroscopy between goalkeepers, defenders, and forwards in our study. These results indicate that all ice hockey players, regardless of playing position, are potential targets for secondary prevention. While forwards are reportedly most frequently injured in the hip and groin region, goalkeepers spend the most time on ice of all playing positions and seem to be at higher risk than other players once this is taken into account [5, 8]. However, it is possible that, due to our small sample of players, we failed to find a statistically significant difference despite an existing difference in the general population of players. Furthermore, the lower response rate for defenders and forwards could have potentially led to an overestimation of problems in this group when compared to goalkeepers, if affected defenders and forwards were more likely to respond to the survey. Results of this study should therefore not be considered to clearly indicate either lower or higher prevalence of problems among goalkeepers. It is plausible, however, that goalkeepers, once they have hip and groin problems, are affected to a larger extent than defenders or forwards, given the high load on the hip and groin region associated with the modern style of goalkeeping [31].

In this study, 26 players (7.9%) reported having had previous hip or groin surgery. The majority of these surgeries were hip arthroscopies for FAIS [*n* = 17 (65%)]. Even though 75% of youth ice hockey players and 70% of professional ice hockey players are reported to have CAM morphologies [18, 21], the majority of players do not develop FAIS, defined by the presence of morphological changes, and clinical signs and symptoms [12]. It has, however, been reported that 1 in 10 hip and groin injuries in the NHL are intra-articular [8] and that arthroscopic treatment of FAIS in ice hockey players is common [19, 22]. Importantly, our results represent players active on an elite level of play, thereby excluding all players that may have ceased their ice hockey participation due to hip problems such as FAIS. Since approximately half of all patients undergoing FAIS surgery are expected to return to their pre-injury level of sport participation [14, 34], the proportion of players reporting history of surgery in this study may underestimate the burden of intra-articular hip problems leading to surgical interventions in ice hockey players.

Players reported hip and groin problems retrospectively. Even though binary questions regarding injury history, as used in this study, can provide valid athlete responses 12 months in retrospect [9], it is possible that players did not accurately report the problems experienced in the previous season. Furthermore, a broad definition of hip and groin problems was applied, potentially including a variety of underlying pathologies. Preferably, the Doha-agreement on terminology and definition of groin pain in athletes would have been used [30]. However, due to the anonymous reporting of injuries by players, clinical examinations and classification were not feasible in this study. It should be acknowledged that the low response rate for players that were indirectly invited to participate in the study may have resulted in a selection bias. It is unknown how many of the players, targeted in this last recruitment step, actually received the survey. Hence, even though the results of this study are in accordance with previous research on different athletic populations [27] they need to be confirmed in future prospective studies.

## Conclusion

Hip and groin problems are prevalent in male ice hockey players, regardless of playing position. Players with a history of hip and groin problems during the previous season had significantly worse hip and groin function at the beginning of the new season and longer symptom durations were associated with more disability. Findings of this study highlight the need to focus on primary and secondary preventive efforts on all players in an ice hockey team.
